# Supplementary material for: kri-1/KRIT1 restrains skn-1/NRF2 activation to promote innate immune and lipid homeostasis
Source: bioRxiv. 2026 May 18:2026.05.15.725342. Preprint. [Version 1] doi: 10.64898/2026.05.15.725342 (PMC13228257; doi:10.64898/2026.05.15.725342)
Supplement: Supplement 1 [file NIHPP2026.05.15.725342v1-supplement-1.pdf]

bioRxiv preprint doi: https://doi.org/10.64898/2026.05.15.725342; this version posted May 18, 2026. The copyright holder for this preprint (which was not certified by peer review) is the author/funder, who has granted bioRxiv a license to display the preprint in perpetuity. It is made available under a CC-BY 4.0 International license.

**Figure S1. *kri-1/KRIT1* functions independently of SKN-1 to support intestinal barrier integrity.** *C. elegans* animals of the indicated genotypes were examined using the “smurf” assay, which assesses the permeability of the intestinal barrier. Percentage of animals exhibiting “non-smurf”, “intermediate”, or “smurf” phenotype in a qualitative assessment is shown. * $p<0.05$ (Fisher’s exact test). Scale bar: 100 μM. Sample sizes and p-values are shown in Table S1.

**Table S1. Source Data, including sample sizes, statistical tests, and p values for all data in this manuscript.**

bioRxiv preprint doi: https://doi.org/10.64898/2026.05.15.725342; this version posted May 18, 2026. The copyright holder for this preprint (which was not certified by peer review) is the author/funder, who has granted bioRxiv a license to display the preprint in perpetuity. It is made available under a CC-BY 4.0 International license.

657 **Table S2. Primer, crRNA guide and ssODN sequences designed for this study**
